# PREDICTIVE MODEL OF 30-DAY HOSPITAL READMISSION FOR PATIENTS WITH ALZHEIMER’S DISEASE

**DOI:** 10.1093/geroni/igac059.722

**Published:** 2022-12-20

**Authors:** Elham Mahmoudi, Cyrus Najarian, Wenbo Wu, James Aikens, Julie Bynum

**Affiliations:** University of Michigan, Ann Arbor, Michigan, United States; University of Michigan, Ann Arbor, Michigan, United States; University of Michigan, Ann Arbor, Michigan, United States; University of Michigan, Ann Arbor, Michigan, United States; University of Michigan, Ann Arbor, Michigan, United States

## Abstract

Hospitals are insufficiently equipped for patients with Alzheimer’s disease and related dementia (ADRD). Thus, 30-day hospital readmission is higher and costlier among ADRD patients than the general population of older adults. Our objective was to develop a risk-assessment tool for hospitalized patients with ADRD. We used 2016-2019 electronic medical record (EMR) data from the University of Michigan health system and applied machine learning techniques to develop a readmission risk-assessment tool. We identified 2,899 individuals with ADRD who had at least one index hospital admission. All data features available in EMR – demographics, lab results, prior counts of healthcare use, and characteristics of index hospitalization – were included in our predictive models. Additionally, we geocoded the street address of patients using the National Neighborhood Data Archive using the U.S. Census tract-level information to include two composite measures of socioeconomic status: disadvantage and affluence. The readmission rate for ADRD patients was 22% versus 17% for the general population. The best predictive model was the Random Forest (area under the receiver operating characteristic curve=0.66; sensitivity=0.64; specificity=0.61). The accuracy of our model (0.61) was 42% higher than the LACE score (0.43), which is currently used by the hospital for all patients. The top 5 predictors of 30-day readmission among people with ADRD included length of hospital stay, frailty index, living in a disadvantaged neighborhood, and total prior-year healthcare charges. Our risk-assessment tool identifies ADRD patients at high risk of readmission and why they are at higher risk. The tool enables better decision-making before discharge.

